# Morphology-tunable and pH-responsive supramolecular self-assemblies based on AB_2_-type host–guest-conjugated amphiphilic molecules for controlled drug delivery

**DOI:** 10.3762/bjoc.15.188

**Published:** 2019-08-13

**Authors:** Yang Bai, Cai-ping Liu, Di Chen, Long-hai Zhuo, Huai-tian Bu, Wei Tian

**Affiliations:** 1 Shaanxi Key Laboratory of Chemical Additives for Industry, College of Chemistry and Chemical Engineering, Shaanxi University of Science and Technology, Xi’an 710021, China; 2 MOE Key Laboratory of Material Physics and Chemistry under Extraordinary Conditions and Shaanxi Key Laboratory of Macromolecular Science and Technology, School of Science, Northwestern Polytechnical University, Xi’an, 710072, China; 3 Institute of Basic Medical Sciences, Xi’an Medical University, Xi’an 710021, China

**Keywords:** β-cyclodextrin, controlled drug delivery, host–guest interaction, stimuli-responsive, supramolecular self-assemblies

## Abstract

Although stimuli-responsive supramolecular self-assemblies have been constructed, the controlled drug delivery induced by morphology transitions of these supramolecular self-assemblies on the basis of host–guest-conjugated monomers (HGCMs) are few reported. In this paper, the self-assembly behaviors of AB_2_-type HGCMs, e.g., β-cyclodextrin-benzimidazole_2_ (β-CD-BM_2_), were investigated at neutral and acidic pH conditions, respectively. Specifically, β-CD-BM_2_ first self-assembled into fan-shaped supramolecular self-assemblies with a hydrodynamic diameter of 163 nm at neutral pH, whereas they were further dissociated into spherical supramolecular self-assemblies with a size of 52 nm under acidic conditions. This morphology transition process was utilized to conduct a two-stage DOX delivery under neutral and acidic pH. Basic cell experiments demonstrated that the drug-loaded β-CD-BM_2_-based supramolecular self-assemblies with varied morphology could inhibit cancer cell proliferation, indicating their potential application in the field of drug delivery.

## Introduction

Supramolecular self-assemblies based on noncovalent interactions with dynamic nature and reversible property have attracted increasing attention in the fields of biomedicine [1–7], smart materials [8–10], etc. As one common noncovalent interaction [11], host–guest interaction has been used to effectively create stimuli-responsive supramolecular self-assemblies with regulated self-assembly morphologies due to their response to various external stimuli, such as temperature [12], light [13–15], redox [16–18], and pH [19–20]. In addition, β-cyclodextrin (β-CD), pillararene and cucurbituril have been utilized as host units to construct these stimuli-responsive supramolecular self-assemblies [21–27]. For example, β-CD can form inclusion complexes with guests such as azobenzene [28–29], ferrocene [30–31] and benzimidazole [32–34] to construct light-, redox-, and pH-responsive supramolecular self-assemblies. In the abovementioned self-assemblies; however, host and guest units have to be synthesized individually, or were incorporated into different moieties or polymer chains as terminal or side groups. Thus, the preparation procedures of the stimuli-responsive supramolecular self-assemblies were fairly complicated, and their self-assembly behaviors could hardly effectively be regulated.

Alternatively, host–guest-conjugated monomers (HGCMs) which gather host and guest units into one molecule have been studied and attracted many attentions in the field of self-assembly [35]. AB-type HGCMs containing one host and other guest moieties have been used to construct intramolecular complexes [36], cyclic oligomers [37], supramolecular polymers [23,38–40], gels [41–42], etc. However, AB-type HGCMs were still limited to obtain non-spherical stimuli-responsive supramolecular self-assemblies with a tunable morphology transition ability. According to the literature [43–44], some nonspherical supramolecular self-assemblies seemed to be more efficient in the cellular internalization. Thus, we intend to design AB_2_-type amphiphilic HGCMs to form nonspherical supramolecular self-assemblies on the basis of their asymmetric host–guest unit number. Thus, an effective and controlled release of drugs might be realized due to tunable morphology transitions and stimuli-responsive properties of supramolecular self-assemblies.

**Scheme 1:**
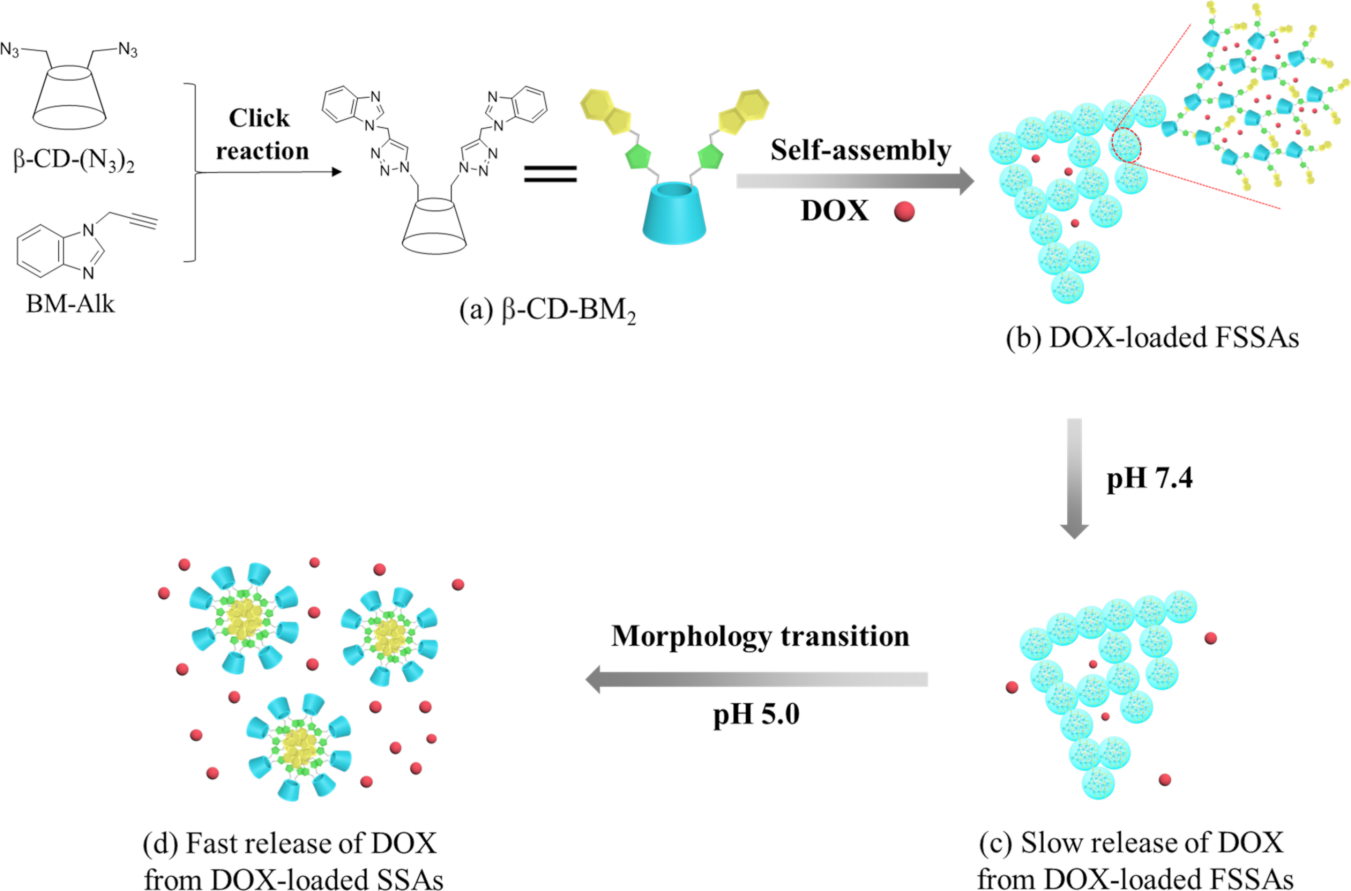
Schematic illustration of the construction of β-CD-BM_2_-based supramolecular self-assemblies, their morphology transitions and drug release behaviors. (a) Chemical structure of β-CD-BM_2_; (b) DOX-loaded fan-shaped self-assemblies (FSSAs); (c) Slow release of DOX from DOX-loaded FSSAs at pH 7.4; (d) Fast release of DOX from DOX-loaded spherical self-assemblies (SSAs) at pH 5.0.

On the basis of the considerations described above, herein we report on pH-responsive supramolecular self-assemblies by utilizing β-CD-benzimidazole_2_ (β-CD-BM_2_) as AB_2_-type amphiphilic HGCMs for the delivery and controlled release of doxorubicin (DOX). β-CD-BM_2_ was first synthesized by click reaction (Scheme 1a). β-CD-BM_2_ formed fan-shaped self-assemblies (FSSAs) at neutral pH through host–guest interactions between β-CD and BM. DOX was used as a model drug encapsulated into FSSAs (Scheme 1a,b). The slow release of DOX from DOX-loaded FSSAs was observed at pH 7.4 (Scheme 1b,c). On the contrary, the release rate of DOX increased evidently when the solution pH value was adjusted to 5.0, accompanied with morphology transitions from FSSAs to spherical self-assemblies (SSAs) due to the pH-induced dissociation of β-CD/BM inclusion complexes (Scheme 1c,d), and the hydrophilic–hydrophobic interaction-induced formation of spherical micelles with BM units as inner hydrophobic core and β-CD moieties as outer hydrophilic shell. The basic cell experiments confirmed that the pH-responsive supramolecular self-assemblies based on β-CD-BM_2_ have a potential application in the field of drug delivery.

## Results and Discussion

### Synthesis of the host–guest-conjugated amphiphilic molecule β-CD-BM_2_

β-CD-N_3_(-OTs) was first prepared according to our previous work [45], and then a substitution reaction was performed to obtain the functionalized β-CD molecule containing two azide groups (β-CD-(N_3_)_2_). The FTIR spectrum of β-CD-(N_3_)_2_ (Figure S1A-b, Supporting Information File 1) showed the appearance of azido absorption peaks at 2103 cm^−1^. Characteristic signals of protons a, b and c in the -OTs group of β-CD-N_3_(-OTs) at δ = 7.4–7.7 and 2.4 disappeared in the ^1^H NMR spectrum (Figure S1B-b, Supporting Information File 1). The above results confirmed that the substitution reaction has been successfully conducted. Subsequently, BM-Alk was synthesized by the alkylation reaction between benzimidazole and propargyl bromide according to the literature [46]. The appearance of the alkynyl absorption peaks at 2127 cm^−1^ in the FTIR spectrum (Figure S1A-a, Supporting Information File 1) and the characteristic signals for BM and propargyl groups in ^1^H NMR spectrum (Figure S1B-c, Supporting Information File 1), accompanied with the GC–MS results (156.1), indicated the successful preparation of BM-Alk.

Finally, the targeted monomer β-CD-BM_2_ was synthesized through the click reaction between β-CD-(N_3_)_2_ and excess BM-Alk (Scheme 1a). As can be seen from the FTIR spectrum of β-CD-BM_2_ in Figure S1A-c (Supporting Information File 1), the absorption peaks of the azido group at 2103 cm^−1^ and the alkynyl group at 2127 cm^−1^ disappeared simultaneously. Characteristic signals for β-CD at δ = 3.1–3.9, 4.3–4.9 and 5.6–5.9 and protons of BM at δ = 7.0–8.4 could be observed obviously in the ^1^H NMR spectrum of β-CD-BM_2_ (Figure S1B-d, Supporting Information File 1). Furthermore, the molecular weight of β-CD-BM_2_ (Figure S1C, Supporting Information File 1) measured by high-resolution mass spectrometry was 1497.5266 [M + H^+^], which was in accordance with the theoretical value of 1496.5236. On the basis of the above results, AB_2_-type host–guest-conjugated amphiphilic monomer β-CD-BM_2_ has been successfully synthesized.

### Supramolecular self-assembly behavior of β-CD-BM_2_

**Figure 1:**
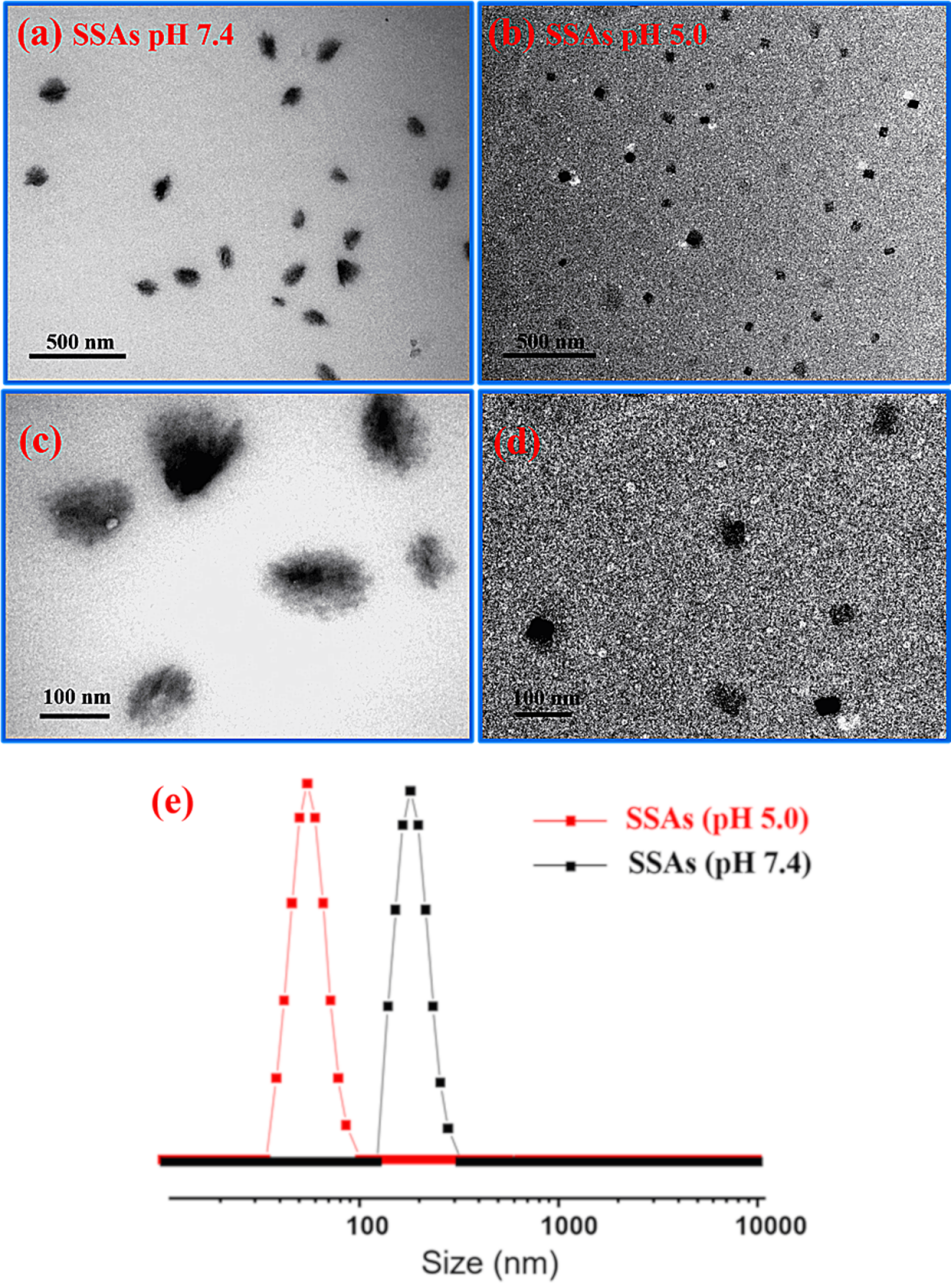
Typical TEM images (a–d) and DLS curves (e) of β-CD-BM_2_-based supramolecular self-assemblies at pH 7.4 (a, c, e) and pH 5.0 (b, d, e), respectively.

The morphology of the self-assembly and the size of β-CD-BM_2_-based supramolecular self-assemblies under neutral and acidic pH were first investigated by employing transmission electron microscopy (TEM) and dynamic light scattering (DLS). The prepared β-CD-BM_2_ were dissolved under neutral pH solution to form FSSAs. TEM revealed that these FSSAs had an average diameter (*D*_av_) of 120 nm (Figure 1a,c), which was close to the hydrodynamic diameter (*D*_h_) of 163 nm determined by DLS (Figure 1e). Furthermore, the pH-induced self-assembly morphology transition process were further studied. When the pH value of the FSSAs solution was adjusted to 5.0, SSAs (Figure 1b,d) with a *D*_av_ of 40 nm and *D*_h_ of 52 nm were formed instead of FSSAs.

**Figure 2:**
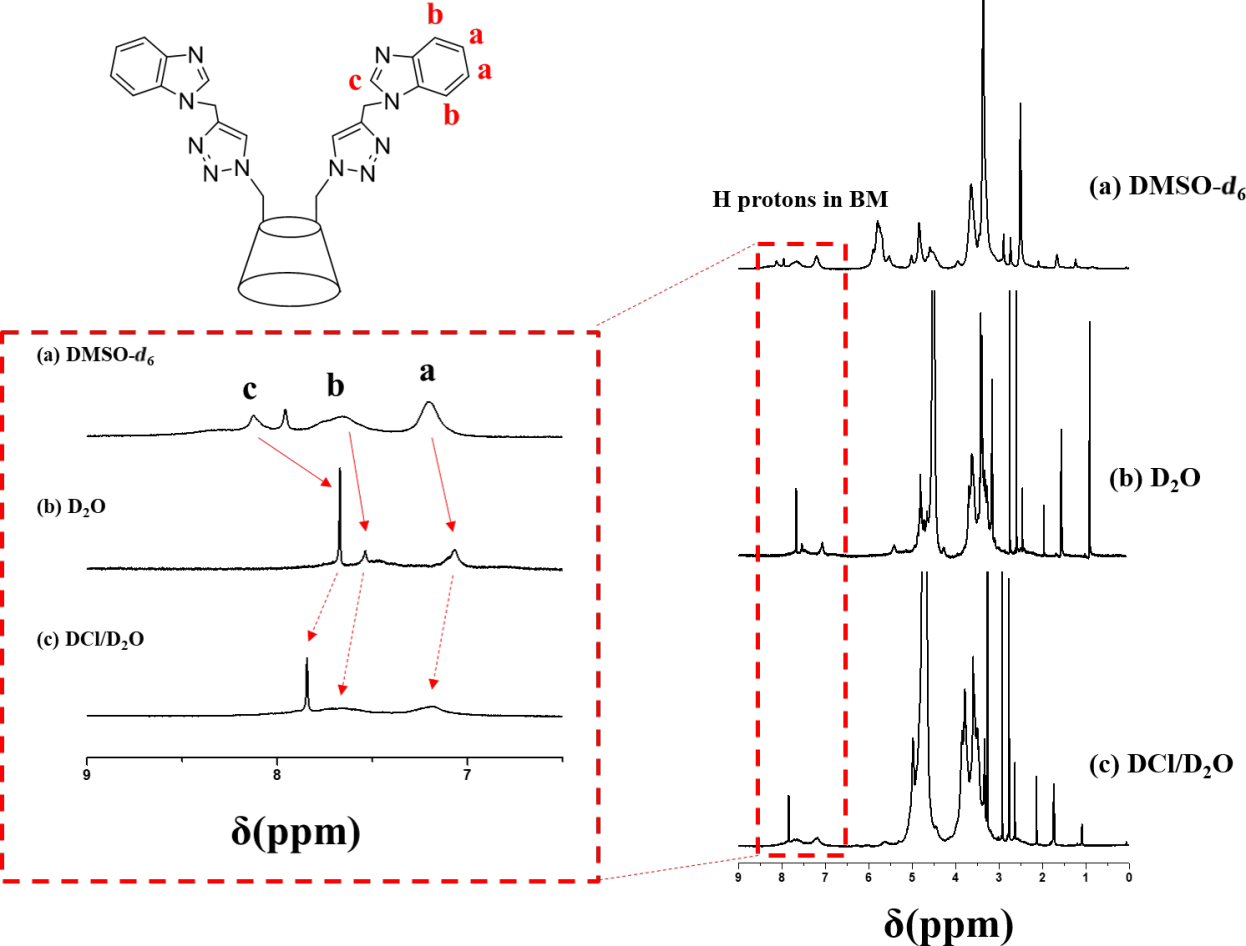
^1^H NMR spectra of β-CD-BM_2_-based supramolecular self-assemblies in DMSO-*d*_6_ (a), D_2_O (b) and DCl/D_2_O (c), respectively.

The ^1^H NMR spectra in D_2_O or D_2_O/DCl was performed to explore the internal structure of supramolecular self-assemblies (Figure 2). As shown in Figure 2b, the proton peak ratios of 2,4,3,5,6-H protons of β-CD to BM protons in D_2_O was 3.75, which is slightly bigger than that of 3.46 in DMSO-*d*_6_ (Figure 2a) due to the shielding effect of host–guest inclusion. The proton peak ratios of 2,4,3,5,6-H protons of β-CD to BM protons in DCl/D_2_O was 24.96 (Figure 2c), which is bigger than that of the ratio of 3.75 in D_2_O (Figure 2b). The evident weakening of BM signals in DCl/D_2_O indicated that the core layer of supramolecular self-assemblies was mainly attributed to the hydrophobic BM moiety. This result revealed that the hydrophobic BM formed the “core”, whereas the hydrophilic β-CD formed the shell layer of SSAs in DCl/D_2_O due to the pH-induced dissociation of β-CD/BM inclusion complexes. In addition, the shift of the BM signals to lower field when the pH value was changed from neutral to acidic pH also confirmed the above result.

**Figure 3:**
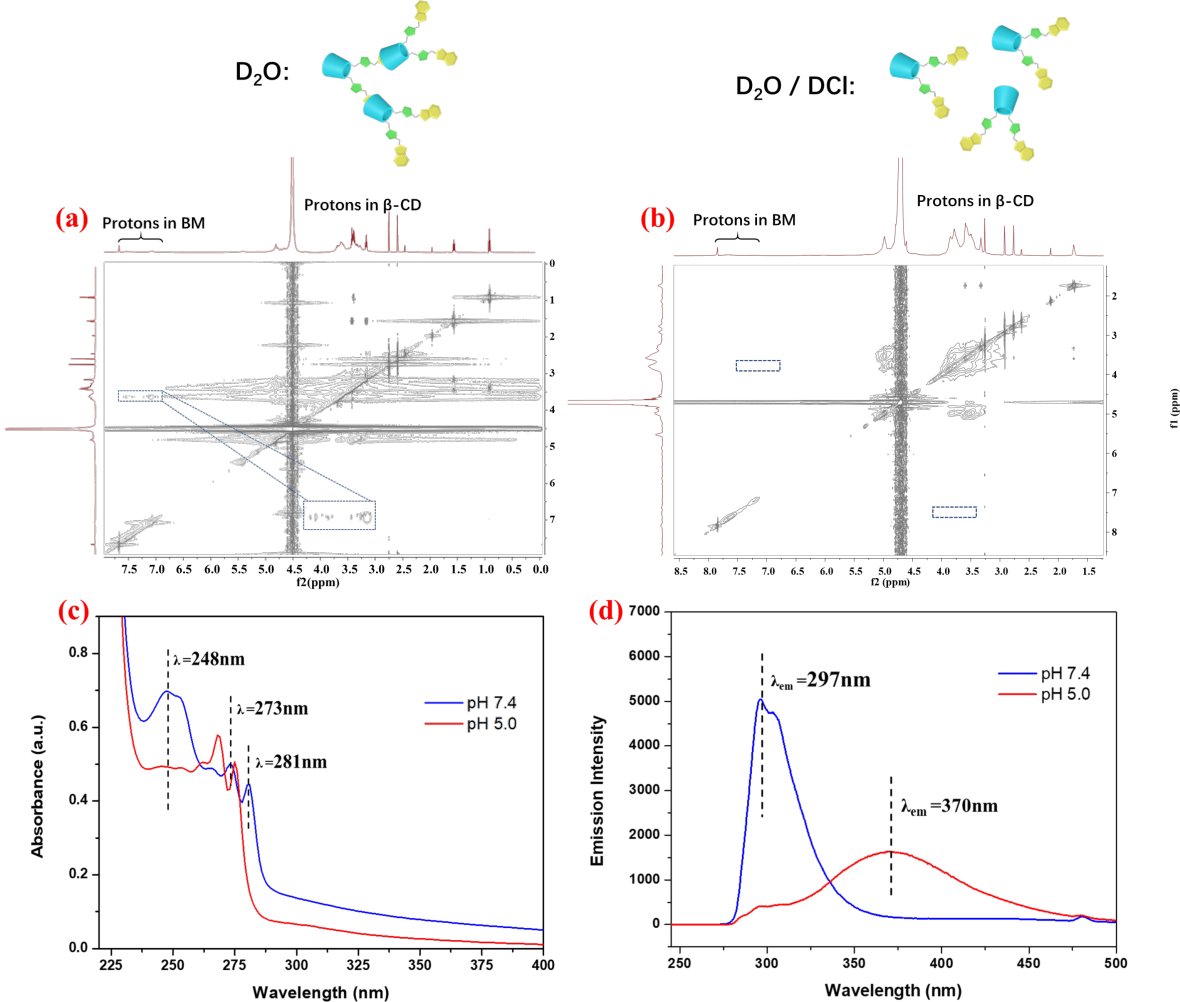
2D NMR NOESY spectra in D_2_O (a) and D_2_O/DCl (b), UV–vis spectra (c) and fluorescence spectra (d) of β-CD-BM_2_-based supramolecular self-assemblies at pH 7.4 and 5.0.

On the basis of the abovementioned results, a possible morphology transition mechanism of β-CD-BM_2_-based supramolecular self-assemblies in different pH solutions was proposed. In neutral aqueous solution, the host–guest interaction between β-CD and BM in β-CD-BM_2_ were enhanced, and further driven by β-CD-BM_2_ to form the FSSAs. While the BM could be protonated in the acidic environment [47], so the host–guest interaction of β-CD and protonated BM decreased correspondingly, resulting in a self-assembly morphology transition from fan-shaped to spherical structures. Furthermore, 2D NOESY, UV–vis and fluorescence spectroscopy were employed to confirm the above proposed mechanism. Firstly, the 2D NOESY spectra of solutions of supramolecular self-assemblies further testified the inclusion interaction between β-CD and unprotonated/protonated BM. As shown in Figure 3a, the signals from the BM protons (H protons of the benzene ring) were correlated with the signals of the inner 3-H and 5-H protons of β-CD in neutral aqueous solution, indicating the formation of the host–guest inclusion complexes between the β-CD and the BM moieties. On the contrary, the 2D NOESY spectra of SSAs showed no correlation peak between the signals of the protons of BM and the inner 3-H and 5-H protons of β-CD at pH 5.0 (Figure 3b). Second, the UV–vis results (Figure 3c) showed that the absorption band at λ = 248 nm decreased and the absorption band at λ = 281, 273 nm shifted to 275, 268 nm when the solution pH was changed from 7.5 to 5.0, owing to the protonation of BM inclusion complexes. In addition, the fluorescence spectra indicated that the maximum emission wavelength shifted from 297 nm at pH 7.4 to 370 nm at pH 5.0 (Figure 3d), indicating as well the protonation of BM inclusion complexes. These results further proved that the self-assembly morphology transitions of supramolecular self-assemblies was driven by the pH-induced dissociation of host–guest interactions between BM and β-CD.

### Controlled release behaviors of drug-loaded supramolecular self-assemblies

**Figure 4:**
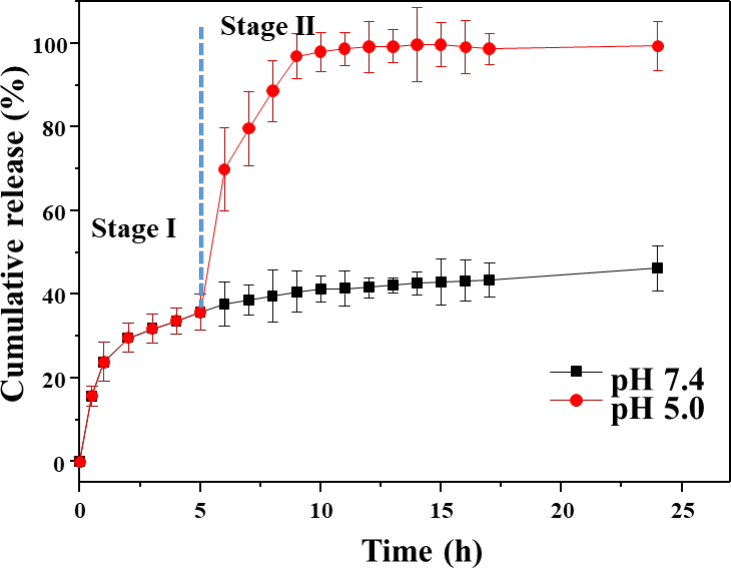
Cumulative release curves of DOX-loaded β-CD-BM_2_ based SSAs at pH 7.4 and 5.0, respectively.

β-CD-BM_2_-based SSAs were used as nanocarriers for drug delivery. Doxorubicin (DOX) was first loaded into FSSAs with a drug-loaded content of 8.2%. To confirm the pH-induced controlled release behavior, a two-stage DOX-release process was conducted at different pH conditions. Release curves of DOX from DOX-loaded FSSAs are shown in Figure 4. The release rate of DOX was suppressed at pH 7.4, and only about 42% of DOX was released within 24 h. In contrast, the release rate of DOX was evidently increased when the solution pH value was changed from 7.4 to 5.0. The cumulative release amounts of DOX correspondingly increased from 42% to 98%. The above result may be attributed to the pH-induced morphology transitions from FSSAs to SSAs on the basis of the dissociation of host–guest interactions between β-CD and BM.

### Cellular toxicity of drug-loaded supramolecular self-assemblies

**Figure 5:**
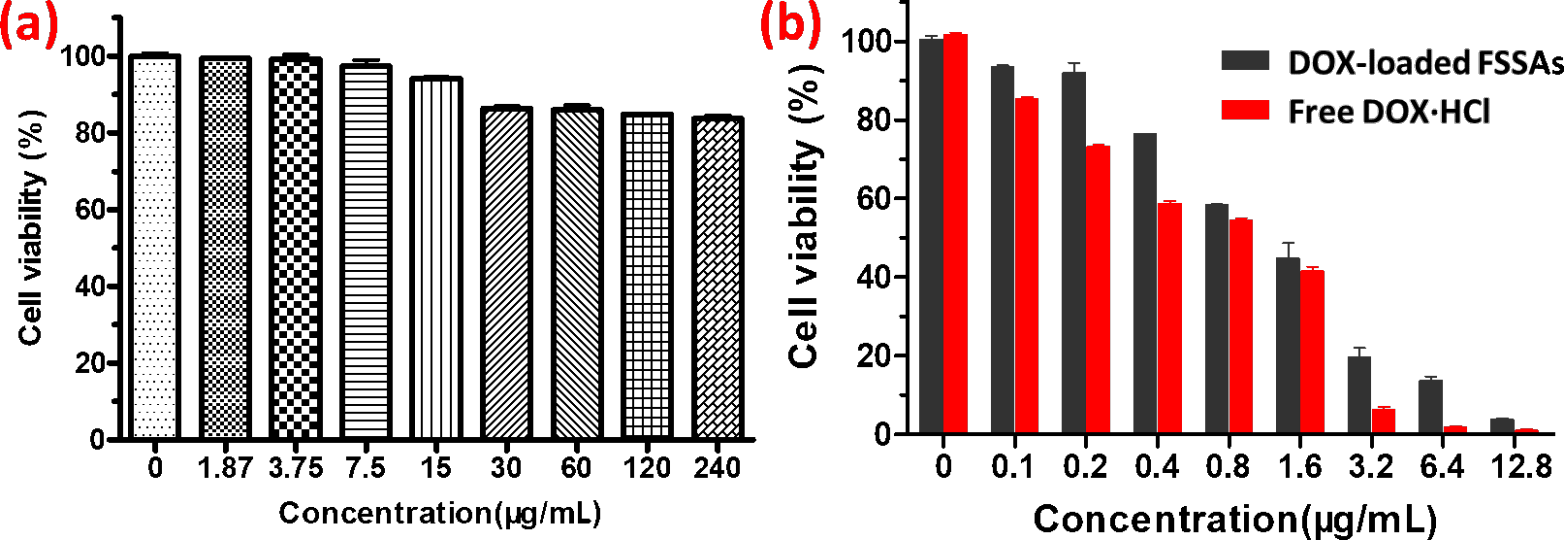
(a) Cell viability of PC-3 cells after incubated with β-CD-BM_2_ based FSSAs for 48 h. (b) In vitro cytotoxicity of DOX-loaded β-CD-BM_2_ based FSSAs and free DOX·HCl against PC-3 cells after incubation for 48 h.

The biocompatibility of drug-free supramolecular self-assemblies is of crucial importance for the further use of these materials as drug carriers. Herein, the biocompatibility of β-CD-BM_2_-based FSSAs towards PC-3 cells was investigated with different concentrations after incubation for 48 h. The result did not show any cytotoxicity against PC-3 cells (Figure 5a). The viability of PC-3 cells could reach 84% even when the concentration of FSSAs was up to 240 μg/mL, indicating a good biocompatibility. An MTT assay was then conducted to evaluate the potential of supramolecular self-assemblies as intelligent drug release carriers within a biological environment. The cellular toxicity of DOX-loaded FSSAs and free DOX·HCl against PC-3 cells was further investigated. As shown in Figure 5b, DOX-loaded FSSAs displayed reduced cytotoxicity against PC-3 cells in comparison with free DOX·HCl. The in vitro half-maximal inhibitory concentration (IC_50_) values of DOX-loaded FSSAs and free DOX·HCl after incubation for 48 h were 1.44 and 0.91 μg/mL, respectively. The results further certified that the fast intracellular drug-release process of DOX-loaded FSSAs provided a large intracellular drug dose and high cytotoxicity. All these results suggested that DOX-loaded FSSAs presented a potential application in controlled drug delivery.

### Intracellular uptake of drug-loaded supramolecular self-assemblies

**Figure 6:**
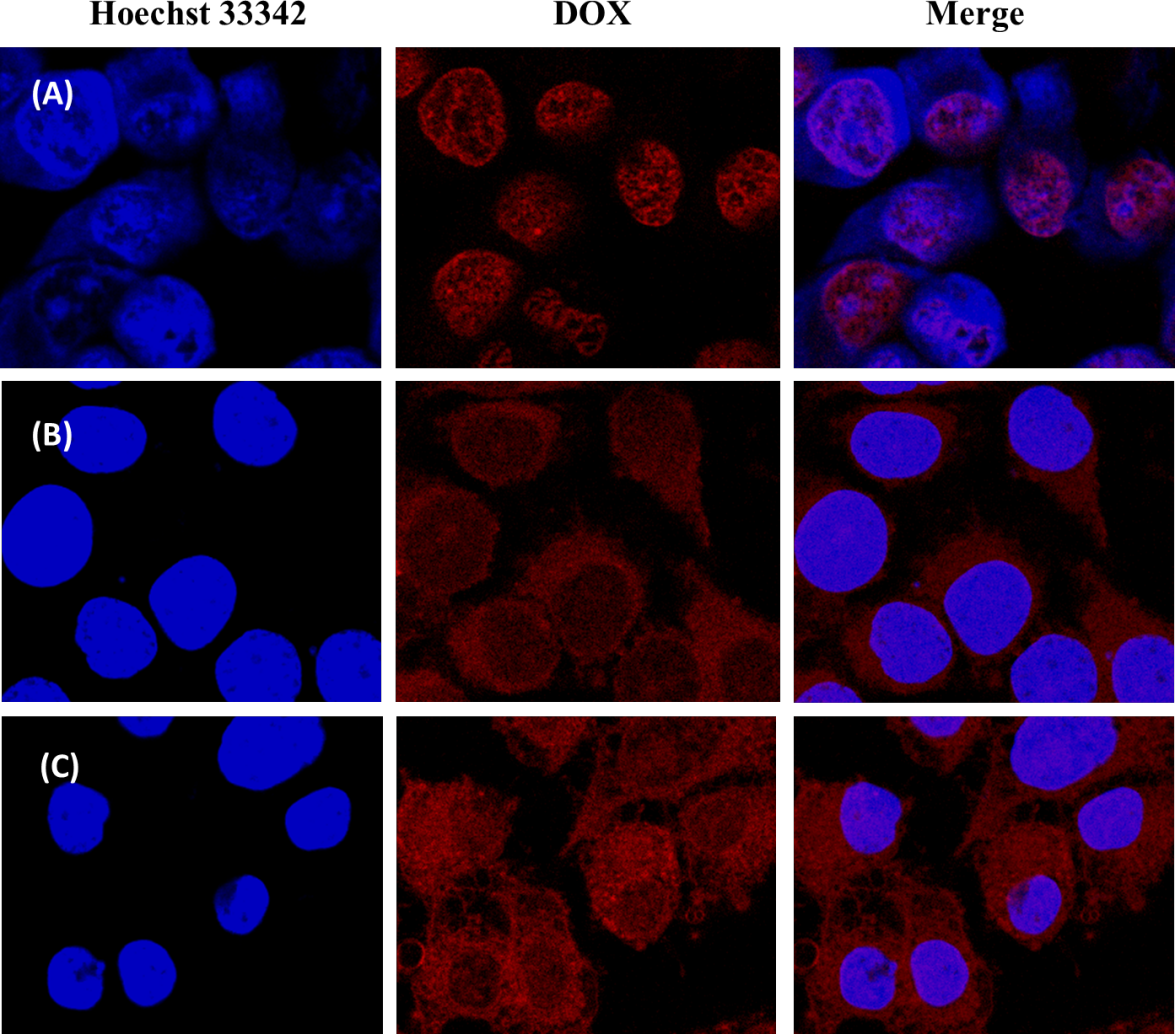
CLSM images of PC-3 cells incubated with the FSSAs and free DOX·HCl at a concentration of 5 μg/mL. From left to right: Hoechst 33342 (blue), DOX (red) and a merge of two images. (A) Free DOX·HCl, 4 h; (B) FSSAs, 1 h; (C) FSSAs, 4 h.

Confocal laser scanning microscopy (CLSM) was further utilized to confirm the intracellular uptake of DOX-loaded FSSAs (Figure 6). The PC-3 cells treated with DOX-loaded FSSAs indicated Hoechst 33342 blue fluorescence in their nuclei and DOX red fluorescence in their cytosol after incubation. Moreover, the intensity of DOX red fluorescence was increased when the incubation time was prolonged from 1 h to 4 h. This result indicated that DOX-loaded FSSAs were internalized into PC-3 cells and DOX could be released from the FSSAs under endosome acidic conditions. Thus, DOX-loaded FSSAs could be considered to deliver and release DOX in cancer cells.

## Conclusion

In summary, an AB_2_-type host–guest-conjugated amphiphilic monomer, β-cyclodextrin-benzimidazole_2_ (β-CD-BM_2_), was successfully prepared to construct pH-responsive supramolecular self-assemblies. On the basis of the pH-induced association and disassociation of β-CD/BM complexes, these supramolecular self-assemblies with adjustable morphology and size were obtained. The fan-shaped supramolecular self-assemblies were first obtained based on the host–guest interaction between β-CD and BM, then further dissociated under acidic conditions into spherical supramolecular self-assemblies with smaller size. The morphology transitions can be utilized to realize a two-stage drug release. The uptake of the DOX-loaded supramolecular self-assemblies performed efficiently and the cellular toxicity through inhibiting cell proliferation was high. All these results indicate that these supramolecular self-assemblies might have a potential application in the field of controlled release.

## Supporting Information

File 1 Experimental section.
