# Supplementary material for: Morphology-tunable and pH-responsive supramolecular self-assemblies based on AB2-type host–guest-conjugated amphiphilic molecules for controlled drug delivery
Source: Beilstein J Org Chem. 2019 Aug 13;15:1925–32. doi: 10.3762/bjoc.15.188 (PMC6720476; doi:10.3762/bjoc.15.188)
Supplement: File 1 — Experimental section. [file Beilstein_J_Org_Chem-15-1925-s001.pdf]

BEILSTEIN JOURNAL OF ORGANIC CHEMISTRY

Supporting Information

for

Morphology-tunable and pH-responsive supramolecular self-assemblies based on AB_2-type host–guest-conjugated amphiphilic molecules for controlled drug delivery

Yang Bai, Cai-ping Liu, Di Chen, Long-hai Zhuo, Huai-tian Bu and Wei Tian

Beilstein J. Org. Chem. **2019,** *15,* 1925–1932. doi:10.3762/bjoc.15.188

Experimental section

License and Terms: This is a supporting information file under the terms of the Creative Commons Attribution License (http://creativecommons.org/licenses/by/4.0). Please note that the reuse, redistribution and reproduction in particular requires that the authors and source are credited.
The license is subject to the *Beilstein Journal of Organic Chemistry* terms and conditions: (https://www.beilstein-journals.org/bjoc)

Materials

Benzimidazole (BM, 99%) was purchased from J&K Chemical, Ltd. Propargyl bromide (80% in tuluene) and doxorubicin hydrochloride (DOX·HCl, 98%) were acquired from Macklin Chemical China. $Cu(PPh_3)_3Br$ (98%) was purchased from Aladdin Reagent. Sodium hydroxide (NaOH), tosyl chloride, sodium azide (NaN_3), potassium carbonate (K_2CO_3), trimethylamine, acetone, acetonitrile were purchased from Sinopharm Chemical Reagent Co. All of the regents above were used as received. Mono-6-deoxy-6-azido-β-CD (β-CD-N_3) was prepared according to the literature [1].

Synthesis of bifunctional derivatives of β-cyclodextrin, β-CD-N_3(-OTs)

β-CD-N_3 (6.270 g, 5.40 mmol) was dissolved in the NaOH solution (1.6 g in 100 mL water, 40 mmol). Then tosyl chloride dissolved in 3 mL acetonitrile was added dropwise in 45 min at 0 °C. The reaction system was stirred at room temperature for 4 h and filtrated, and the pH value of filtrate was adjusted to neutral and crystallized at 4 °C. The crude crystals were purified through recrystallization in water twice to obtain a white solid (yield: 21.1%). ^{1}H NMR (DMSO-d_6, TMS): δ=2.4 (3H, -CH_3); 3.1-3.9 (2,3,4,5,6-H in *β*-CD); 4.2–4.8 (H-1,6-OH in *β*-CD); 5.6–5.9 (2,3-OH in *β*-CD); 7.44-7.74 (4H, in Ar).

Synthesis of disubstituted derivatives of β-cyclodextrin, β-CD-$(N_3)_2$

β-CD-N_3(-OTs) (500 mg, 0.37 mmol) and NaN_3 (200 mg, 3.1 mmol) were dissolved in 5 mL water. The reaction system was stirred at 80 °C for 24 h and filtrated, then the filtrate was precipitated in excess acetone. The precipitate was washed with a mixed solvent composed of acetone and water (3:1, v/v) for 3 times and dried in vacuum to obtain white solid (yield: 74.3%). FT-IR (KBr): 3354cm^{-1} (v, O-H); 2927cm^{-1} (v, C-H)); 2103cm-1 (v, –N_3). ^{1}H NMR (DMSO-d_6, TMS): δ=3.1-3.9 (2,3,4,5,6-H in *β*-CD); 4.2–4.8 (H-1,6-OH in *β*-CD); 5.6–5.9 (2,3-OH in *β*-CD).

Synthesis of alkynyl-terminated benzimidazole, BM-Alk

A mixture of benzimidazole (0.8 g, 6.77 mmol), K_2CO_3 (1.8685 g, 13.54 mmol) and 3 mL dry DMF was bubbled with nitrogen for 15 min. Then, propargyl bromide (1.208 g, 8.12 mmol) was added and bubbled with nitrogen for another 15 min. The reaction system was stirred at 60 °C for one night and filtrated, the filtrate was concentrated in vacuum to remove solvent, and the residue was purified by column chromatography with ethyl acetate as the eluent to obtain brown oil product. FT-IR (KBr): 3288cm^{-1} (v, N-H); 2127cm^{-1} (v,C≡CH). ^{1}H NMR ($CDCl_3$, TMS): δ=2.5 (1H, ≡C*H*); δ=4.9 (2H, -CH_2-); δ=7.2-7.8 (4H, in Ar); δ=8.0–8.1 (1H, -N=C*H*-). MS (EI): m/z (%) 156.1 (100%).

Synthesis of AB_2 type monomer, β-CD-BM_2

The AB_2 type monomer β-CD-BM_2 was synthesized using the azide-alkyne cycloaddition click reaction. Firstly, a mixture of β-CD-$(N_3)_2$ (36.8 mg, 0.2 mmol), BM-Alk (68.64 mg, 0.44 mmol) and 1.5 mL dry DMF was bubbled with nitrogen for 15 min. Then, $Cu(PPh_3)_3Br$ (37.2 mg) was added and bubbled with nitrogen for another 30 min and sealed under N_2 atmosphere. The reaction system was stirred at 60 °C for 24 h and the resulting solution was poured into an excess of acetone. The collected precipitate was repeatedly dissolved in DMF and precipitated with acetone. The product was dried under vacuum at 30 °C for 3 days (yield :76.9%).

Characterization

Fourier transform infrared (FTIR) spectra were recorded on a VECTOR-22 IR spectrometer, casting samples into thin films on KBr. Transition mode was used and the wavenumber range was set from 4000 cm^{-1} to 400 cm^{-1}.The ^{1}H NMR, ^{13}C NMR spectra and 2D NOESY (nuclear overhauser enhancement spectroscopy) spectra were obtained from a Bruker AV-400 spectrometer (Bruker BioSpin, Switerland), which operates at 400 MHz (^{1}H) in $CDCl_3$, DMSO-d_6, D_2O or DCl. Electrospray ionization mass spectrometry was recorded using a microTOF-QII 10280 (Varian Inc., USA).

Preparation of supramolecular self-assemblies using β-CD-BM_2 as a monomer

In a typical experiment, 10 mg *β*-CD-BM_2 was dissolved in 2 mL DMF and stirred for 5 h at room temperature. And then 2 mL deionized water was added dropwise. After stirring for another 12 h, the mixture was dialysed against water for 2 days (cut-off M_n = 1000) to obtain supramolecular self-assemblies solution.

Investigation on the morphology and size of supramolecular self-assemblies

The size and morphology of the self-assemblies under different pH were revealed by TEM (FEI Tecnai G2 F20 S-TWIN, 150 kV). Samples were prepared by dropping 10 μL of solutions on copper grids without staining. The DLS measurements were performed on a Brookhaven 90Plus Zeta Dynamic Light Scattering Instrument. The light source was a He-Ne laser operating at 632 nm at an angle of 90° (25 °C). Samples were placed in the cell for at least 3 min prior to the measurement to allow for thermal equilibration. Fluorescence emission spectroscopy was employed with a Hitachi F-7000. UV–vis spectrophotometer measurement was performed on Shimadzu UV-2600 spectroscopy.

In vitro release study of supramolecular self-assemblies

The dialysis method was the main way to realize the DOX release study in vitro. The study was performed in PBS of different pH values. An amount of 15

mg AB_2-type monomer β-CD-BM_2 and 2.5 mg DOX·HCl were dissolved in 3 mL DMF, then 40 μL trimethylamine was added. After stirring for 2 h at room temperature, 3 mL deionized water was added dropwise, and then stirring for another 12 h. The mixture was dialyzed against water for 24 h (cut-off M_n = 1000) to remove free DOX and DMF. The resulting DOX-loaded SSAs solution was lyophilized for next use.

The drug release behaviors were investigated as follows. 1.5 mg DOX-loaded SSAs dissolved in 2 mL phosphate-buffered solutions (PBS) at 37 ℃ under pH 7.4 were transferred into dialysis bags (cut-off M_n = 1000). And then the dialysis bags were soaked in a glass bottle containing 40 mL of PBS at 37 °C under constant shaking. At each predetermined time point, 3 mL of the release medium was withdrawn from the bottle for characterization. Meanwhile, the same volume of fresh PBS was added to ensure the total volume. The amount of DOX in each sample was evaluated by UV-2600 spectroscopy (Shimadzu, Japan). For the controlled drug release experiments, the dialysis bags were then transferred to PBS (pH 5.0) after 5 h. The cumulative release of DOX was calculated by using Equation (1) as follows:

$$\text{Cumulative release}(\%) = \frac{100 \times (40.0C_n + 3.0\sum C_{n-1})}{W_0} \qquad (1)$$

In this equation, W_0 (mg) is the weight of the drug in the SHPs; C_n (mg/mL) is the concentration of DOX in the buffer solution withdrawn n times, C_{n-1} (mg/mL) is the concentration of DOX in the buffer solution withdraw $n - 1$ times.

Cell experiments

The PC-3 cell line was purchased from ATCC (American Type Culture Collection, VA). The cells were maintained in DMEM supplemented with 10% FBS, 25 mM 4-(2-hydroxyethyl)-1-piperazineethanesulfonic (HEPES) acid buffer, 100 U/mL penicillin, and 100 μg/mL streptomycin in a humidified atmosphere of 5% CO_2 at 37 °C. The cellular toxicity of the SSAs, DOX-loaded SSAs, free DOX·HCl were tested as follows. Briefly, PC-3 cells were seeded in 96-well plates with a density of 5×10^3 cells per well and incubated overnight. The cells were later incubated for 48 h with a series of varying concentrations of DOX-loaded SSAs, SSAs and free DOX·HCl. After that step, the cytotoxicity was evaluated by adding a MTT (10 μL) solution to each well of the plate. After incubation for 1 h, the absorbance was measured at 490 nm using a Multiskan MK3 Microplate Reader (Thermo Scientific, USA).

For the in vitro cellular uptake study, PC-3 cells were seeded in glass-base dishes at a density of 2×10^5 per dish and incubated overnight at 37 °C. Next, the cells were incubated with DOX-loaded SSAs that had an equivalent concentration of DOX (5 μg/mL) for 1 h or 4 h at 37 °C. The cells were washed thrice with ice-cold PBS and fixed with 4% (w/w) formaldehyde solution for 15 min. The formaldehyde solution was removed, and the cells were further washed thrice with ice-cold PBS. The nuclei were stained with Hoechst 33342 for 10 min at 37 °C. The cells were finally washed thrice with PBS and

transferred into glass-base dishes and observed by a confocal laser scanning microscopy (TCS-SP5, Leica, Germany).

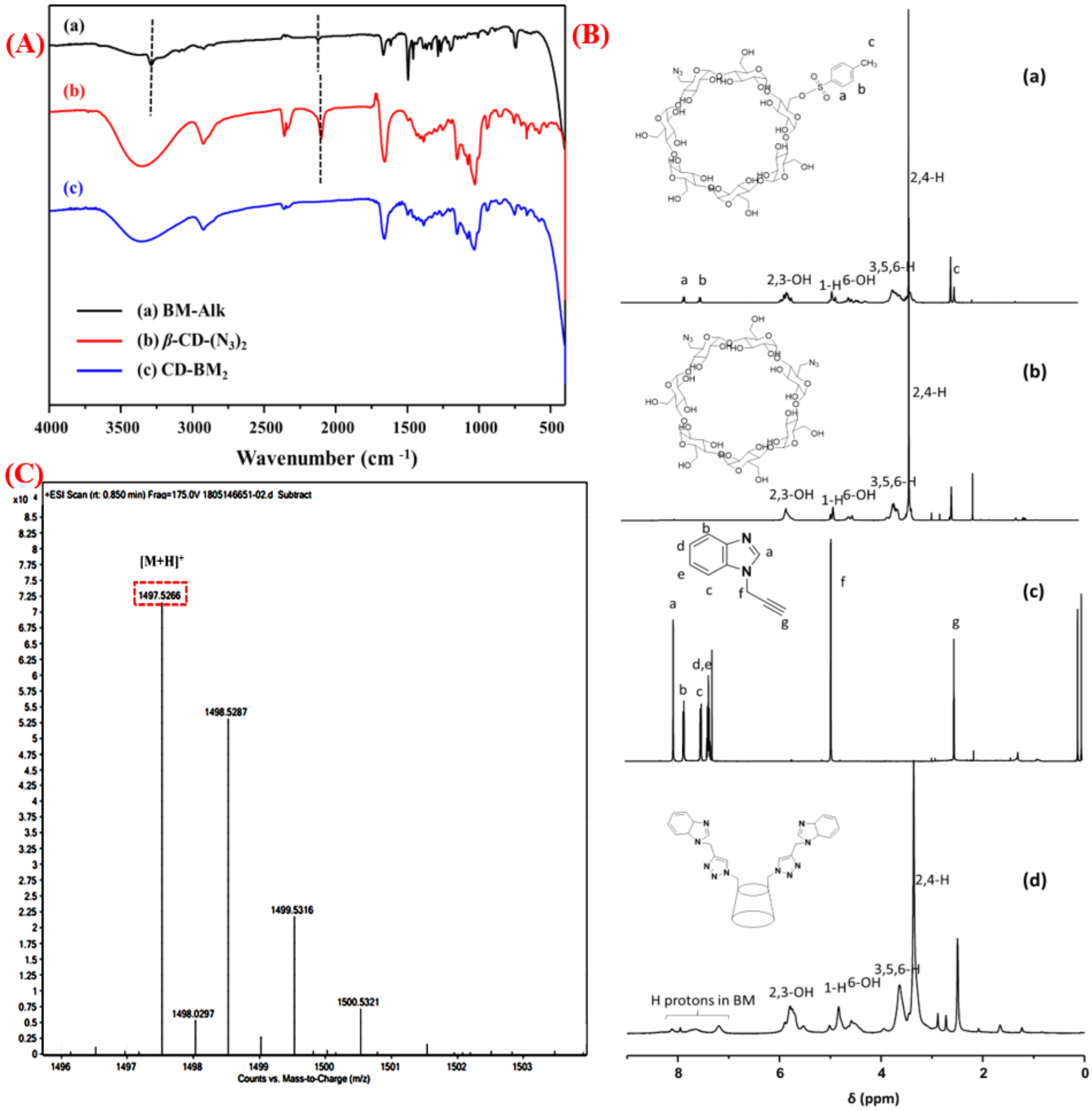

Figure S1: FTIR spectra (A: BM-Alk (a), β-CD-$(N_3)_2$ (b) and β-CD-BM_2 (c)), ^{1}H NMR spectra (B: β-CD-N_3(-OTs) (a), β-CD-$(N_3)_2$ (b), BM-Alk (c) and β-CD-BM_2 (d)) and ESIMS spectrum (C; β-CD-BM_2. The $[M + H]^+$ peak was found at *m*/*z* = 1497.5266, which is consistent with the calculated value *m*/*z* = 1496.5236.)

References

1. Bai, Y.; Fan, X. D.; Tian, W.; Yao, H.; Zhuo, L. H.; Zhang, H. T.; Fan, W. W.; Yang, Z.; Zhang, W. B. *Polymer,* **2013**, *54*, 3566–3573. doi: 10.1016/j.polymer.2013.05.042
